# Combination of neuron-specific enolase measurement and initial neurological examination for the prediction of neurological outcomes after cardiac arrest

**DOI:** 10.1038/s41598-021-94555-0

**Published:** 2021-07-23

**Authors:** Jae Hoon Lee, Yong Hwan Kim, Jun Ho Lee, Dong Woo Lee, Seong Youn Hwang, Chun Song Youn, Ji-Hoon Kim, Min Seob Sim, Kyung Woon Jeung

**Affiliations:** 1 Department of Emergency Medicine, Dong-A University College of Medicine, Busan, South Korea; 2 Department of Emergency Medicine, Samsung Changwon Hospital, Sungkyunkwan University School of Medicine, 158, Paryong-ro, Masanhoewon-gu, Changwon-si, Gyeongsangnam-do 630-723 South Korea; 3 Department of Emergency Medicine, Seoul St. Mary’s Hospital, College of Medicine, The Catholic University of Korea, Seoul, South Korea; 4 Department of Emergency Medicine, Bucheon St. Mary’s Hospital, College of Medicine, The Catholic University of Korea, Seoul, South Korea; 5 Department of Emergency Medicine, Samsung Medical Center, Sungkyunkwan University School of Medicine, Seoul, South Korea; 6 Department of Emergency Medicine, Chonnam National University, Chonnam National University Hospital, Gwangju, South Korea

**Keywords:** Medical research, Neurology

## Abstract

This study aimed to investigate the efficacy of the combination of neuron-specific enolase (NSE) measurement and initial neurological examination in predicting the neurological outcomes of patients with cardiac arrest (CA) by retrospectively analyzing data from the Korean Hypothermia Network prospective registry. NSE levels were recorded at 48 and 72 h after CA. The initial Full Outline of UnResponsiveness (FOUR) and Glasgow Coma Scale (GCS) scores were recorded. These variables were categorized using the scorecard method. The primary endpoint was poor neurological outcomes at 6 months. Of the 475 patients, 171 (36%) had good neurological outcomes at 6 months. The areas under the curve (AUCs) of the categorized NSE levels at 72 h, GCS score, and FOUR score were 0.889, 0.722, and 0.779, respectively. The AUCs of the combinations of categorized NSE levels at 72 h with categorized GCS scores and FOUR score were 0.910 and 0.912, respectively. Each combination was significantly higher than the AUC value of the categorized NSE level at 72 h alone (with GCS: p = 0.015; with FOUR: p = 0.026). Combining NSE measurement and initial neurological examination improved the prediction of neurological outcomes.

## Introduction

Many prognostication tools have been developed to predict the neurological state of patients with comatose mental status after out-of-hospital cardiac arrest (OHCA). However, no single test has an accuracy of 100%. Serological testing is a cheaper, easier, and more rapid prognosticator than imaging or electroencephalography (EEG) and benefits from not being influenced by the required administration of sedatives to patients^1^. The neuron-specific enolase (NSE) assay is the most promising and extensively studied serological test^2^. The increased level of NSE in comatose post-cardiac arrest patients with or without targeted temperature management (TTM) is associated with poor prognosis^3,4^. International guidelines suggest that NSE level alone should not be used to predict poor neurological outcomes because of the possibility of high false-positive rates^5^. If the NSE level alone is unreliable, it appears reasonable to use it in conjunction with another neurological test. Although several studies have reported attempts to combine NSE with an additional prognosticator for the improvement of diagnostic accuracy, it is unclear as to which prognosticator is best combined with NSE.

The Glasgow Coma Scale (GCS) and Full Outline of UnResponsiveness (FOUR) scale are well-known neurological grading scales that are essential for the examination of unconscious patients^6^. In previous studies, the combination of initial neurological examination and other prognostic tools showed better performance than either test alone in predicting neurological outcomes after OHCA^6–9^. However, the potential benefits accruing from a combination of initial neurological examination and the NSE assay have not been fully addressed. As the cutoff NSE level that is predictive of poor outcomes varies across studies, it might be difficult to employ specific cutoff NSE levels to evaluate the dichotomized prognosis of post-cardiac arrest patients and to combine them with other prognosticators^2,4,5,10–15^. Considering these limitations, it might be a good alternative to convert the continuous variables into ordered categories and then assign them differentiated scores. Several scoring systems based on this concept have been developed to predict neurological outcomes after cardiac arrest^6,16^. This study aimed to investigate whether the combination of initial neurological examination and the NSE assay using a scorecard method could improve the prediction of neurological outcomes in patients with OHCA.

## Results

**Figure 1:**
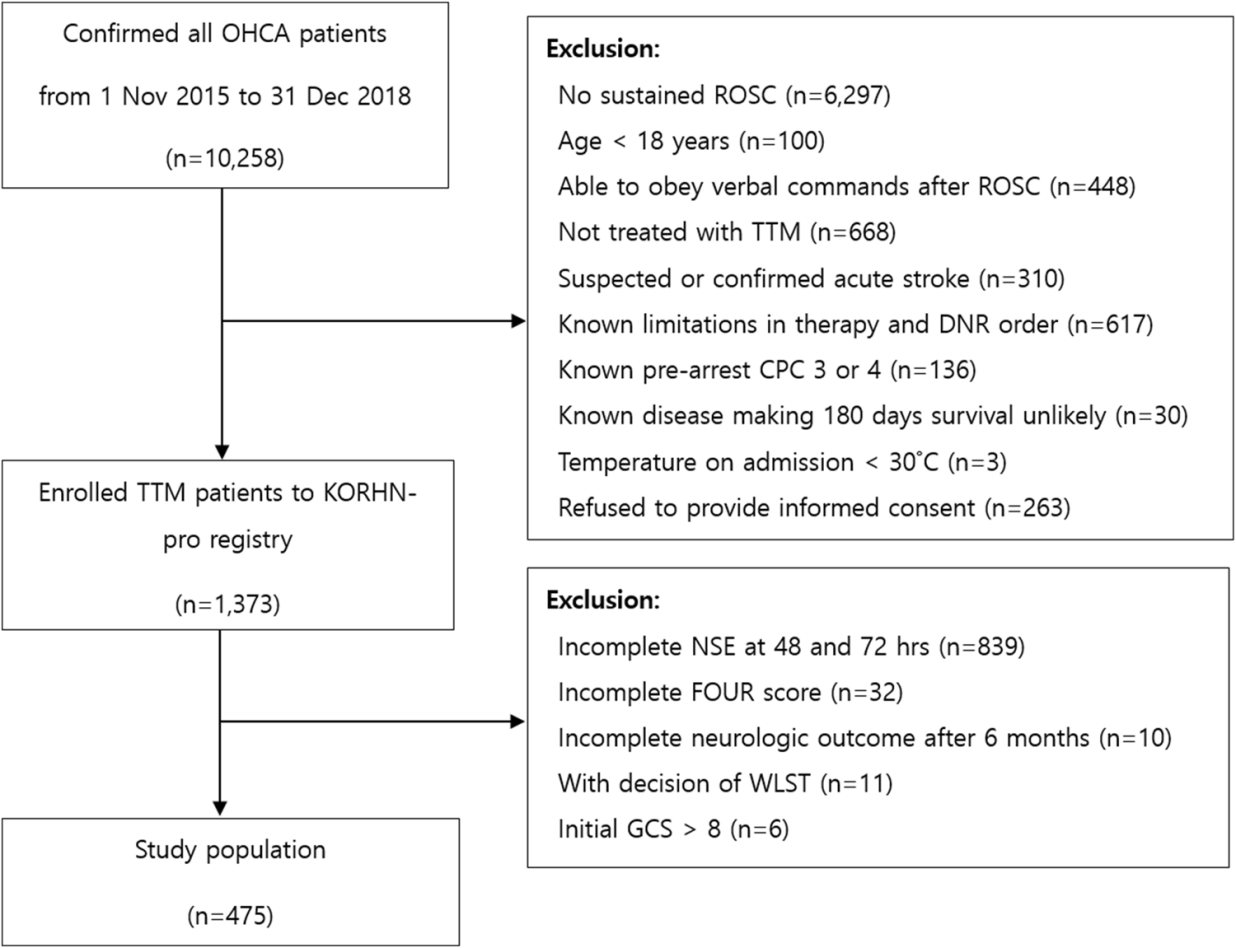
Flow chart depicting the patient selection process. OHCA: out-of-hospital cardiac arrest; ROSC: return of spontaneous circulation; TTM: targeted temperature management; DNR: do not resuscitate; CPC: cerebral performance category; NSE: neuron-specific enolase; FOUR: Full Outline of UnResponsiveness; WLST: withdrawal of life-sustaining therapy; GCS: Glasgow Coma Scale.

Of the 10,258 patients with OHCA, 1373 had their data recorded in the Korea Hypothermia Network prospective (KORHN-pro) registry. Of the 1373 patients, 898 patients were excluded for the following reasons: incomplete NSE-level data at 48 and 72 h after return of spontaneous circulation (ROSC; n = 839); incomplete FOUR scores after ROSC (n = 32); incomplete data concerning neurological outcomes at 6 months (n = 10); withdrawal of life-sustaining therapy (WLST) decision (n = 11); and initial GCS score > 8 (n = 6). The remaining 475 patients were eligible for participation in this study (Fig. 1).

Of the 475 patients, 171 (36%) had good neurological outcomes 6 months after ROSC. The median age of patients was 59 (interquartile range [IQR], 48–69) years, and 72.8% (346/475) of the patients were men. Analgesics, sedatives, and neuromuscular blocking agents were administered to 77.3% (367/475), 90.5% (430/475), and 85.9% (408/475) of patients, respectively. In the study population, the following tests were used for prognostication: brain computed tomography (CT), 453/475 (95.4%); measurement of somatosensory evoked potentials, 186/475 (39.2%); EEG, 297/475 (62.5%); and brain magnetic resonance imaging, 272/475 (57.3%).

Table 1 shows the demographic and clinical features of the patients stratified according to their neurological outcomes. Following ROSC, the total GCS score and FOUR score were higher in the good outcome group than in the poor outcome group (p < 0.001 for both scores). NSE levels at 48 and 72 h were higher in patients with poor outcomes than in those with good outcomes (p < 0.001 for both time points).

**Table 1:** Baseline characteristics and neurological outcomes 6 months after out-of-hospital cardiac arrest.

Variable	Good outcome (n = 171)	Poor outcome (n = 304)	p-value
Age (years)	54 (44–62)	61 (51–71)	< 0.001
Sex, male	132 (77.2)	214 (70.4)	0.110
Initial shockable rhythm	114 (66.7)	50 (16.4)	< 0.001
Prehospital defibrillation	116 (67.8)	67 (22.0)	< 0.001
Witnessed arrest	141 (82.5)	191 (62.8)	< 0.001
Bystander CPR, yes	117 (68.4)	174 (57.2)	0.016
Cardiac etiology	152 (88.9)	146 (48.0)	< 0.001
Cardiac arrest at public place	99 (57.9)	133 (43.8)	0.003
Time from collapse to ROSC, minutes	19 (13–30)	34 (22–45)	< 0.001
Target temperature, < 35 °C	128 (74.9)	234 (77.0)	0.602
Initial lactate level, mmol/L	6.8 (4.1–10.4)	10.0 (4.6–13.4)	< 0.001
**Total FOUR score**	4 (2–7)	1 (0–2)	< 0.001
FOUR—eye response	0 (0–0)	0 (0–0)	< 0.001
FOUR—motor response	1 (0–2)	0 (0–0)	< 0.001
FOUR—brainstem reflex	2 (0–4)	0 (0–1)	< 0.001
FOUR—respiration pattern	1 (0–1)	0 (0–1)	< 0.001
**Total GCS**	4 (3–6)	3 (3–3)	< 0.001
GCS—eye opening	1 (1–1)	1 (1–1)	< 0.001
GCS—verbal response	1 (1–1)	1 (1–1)	0.267
GCS—motor response	2 (1–3)	1 (1–1)	< 0.001
NSE level at 48 h, ng/mL	22 (14–32)	108 (48–215)	< 0.001
NSE level at 72 h, ng/mL	18 (12–27)	139 (55–295)	< 0.001
**CPC at 6 months**			< 0.001
1	153 (89.5)	0 (0)	
2	18 (10.5)	0 (0)	
3	0 (0)	22 (7.2)	
4	0 (0)	40 (13.2)	
5	0 (0)	242 (79.6)	

Data are expressed as n (%) or medians (interquartile ranges). CPR: cardiopulmonary resuscitation; GCS: Glasgow Coma Scale; IQR, interquartile range; ROSC: return of spontaneous circulation; FOUR: Full Outline of UnResponsiveness; NSE: neuron-specific enolase; CPC: cerebral performance category.

The results of the multivariate analyses are shown in Table 2. Each model confirmed that the NSE levels at 48 and 72 h after ROSC, GCS score, and FOUR score were independently associated with poor neurological outcomes at 6 months.

**Table 2:** Multivariate analysis of poor neurological outcomes at 6 months after out-of-hospital cardiac arrest.

	Coefficient	Odds ratio	95% confidence interval		p-value
			Low	Upper	
**Model I**					
Age, years	0.055	1.056	1.030	1.082	< 0.001
Female	0.243	1.276	0.602	2.703	0.526
Initial non-shockable rhythm	1.399	4.051	1.321	12.423	0.014
No prehospital defibrillation	0.142	1.153	0.376	3.533	0.803
Unwitnessed arrest	0.089	1.093	0.471	2.532	0.836
No bystander CPR	0.358	1.431	0.728	2.809	0.299
Non-cardiac etiology	1.024	2.784	1.149	6.745	0.023
Cardiac arrest at non-public place	0.013	1.013	0.520	1.972	0.970
Time from collapse to ROSC, minutes	0.001	1.001	0.982	1.020	0.936
Target temperature ≥ 35 °C	− 0.947	0.388	0.173	0.869	0.021
Initial lactate level, mmol/L	0.050	1.051	0.989	1.117	0.107
GCS score	0.383	1.466	1.124	1.912	0.005
NSE level at 48 h, ng/mL	0.049	1.051	1.035	1.067	< 0.001
**Model II**					
Age, years	0.054	1.055	1.029	1.082	< 0.001
Female	0.199	1.221	0.572	2.604	0.606
Initial non-shockable rhythm	1.376	3.958	1.298	12.066	0.016
No prehospital defibrillation	0.235	1.265	0.416	3.846	0.678
Unwitnessed arrest	0.011	1.011	0.435	2.353	0.979
No bystander CPR	0.497	1.645	0.824	3.279	0.158
Non-cardiac etiology	0.985	2.678	1.102	6.510	0.030
Cardiac arrest at non-public place	0.027	1.027	0.525	2.008	0.938
Time from collapse to ROSC, minutes	0.000	1.000	0.981	1.019	0.986
Target temperature ≥ 35 °C	− 0.825	0.438	0.195	0.983	0.045
Initial lactate level, mmol/L	0.049	1.050	0.988	1.117	0.118
FOUR score	0.209	1.233	1.086	1.401	0.001
NSE level at 48 h, ng/mL	0.049	1.050	1.034	1.066	< 0.001
**Model III**					
Age, years	0.056	1.058	1.030	1.086	< 0.001
Female	0.316	1.372	0.627	3.003	0.429
Initial non-shockable rhythm	1.066	2.904	0.931	9.059	0.066
No prehospital defibrillation	0.533	1.703	0.547	5.301	0.358
Unwitnessed arrest	0.303	1.355	0.545	3.367	0.514
No bystander CPR	0.406	1.502	0.747	3.021	0.254
Non-cardiac etiology	1.137	3.116	1.261	7.700	0.014
Cardiac arrest at non-public place	0.091	1.096	0.549	2.188	0.796
Time from collapse to ROSC, minutes	0.004	1.004	0.983	1.025	0.719
Target temperature ≥ 35 °C	− 0.682	0.505	0.223	1.146	0.102
Initial lactate level, mmol/L	0.016	1.016	0.948	1.089	0.649
GCS score	0.397	1.486	1.138	1.942	0.004
NSE level at 72 h, ng/mL	0.051	1.052	1.036	1.068	< 0.001
**Model IV**					
Age, years	0.055	1.057	1.029	1.085	< 0.001
Female	0.246	1.279	0.586	2.793	0.537
Initial non-shockable rhythm	1.001	2.720	0.893	8.284	0.078
No prehospital defibrillation	0.611	1.842	0.604	5.612	0.283
Unwitnessed arrest	0.261	1.299	0.523	3.226	0.573
No bystander CPR	0.504	1.656	0.819	3.344	0.161
Non-cardiac etiology	1.135	3.111	1.260	7.684	0.014
Cardiac arrest at non-public place	0.122	1.129	0.562	2.268	0.733
Time from collapse to ROSC, minutes	0.002	1.002	0.981	1.021	0.876
Target temperature ≥ 35 °C	− 0.578	0.561	0.247	1.273	0.167
Initial lactate level, mmol/L	0.017	1.017	0.948	1.092	0.633
FOUR score	0.198	1.220	1.074	1.383	0.002
NSE level at 72 h, ng/mL	0.050	1.051	1.035	1.067	< 0.001

CPR: cardiopulmonary resuscitation; GCS: Glasgow Coma Scale; FOUR: Full Outline of UnResponsiveness; NSE: neuron-specific enolase; ROSC: return of spontaneous circulation.

Supplementary Table S1 shows the associations between the 6-month neurological outcomes and strata of NSE level at 48 and 72 h, GCS score, and FOUR score. The proportion of patients with poor 6-month neurological outcomes increased as the NSE level increased at 48 and 72 h. The proportion of patients with poor 6-month neurological outcomes decreased as the GCS score and FOUR score increased. The weighted scores applied to each category of NSE levels at 48 and 72 h, GCS score, and FOUR score are shown in Supplementary Table S2.

**Figure 2:**
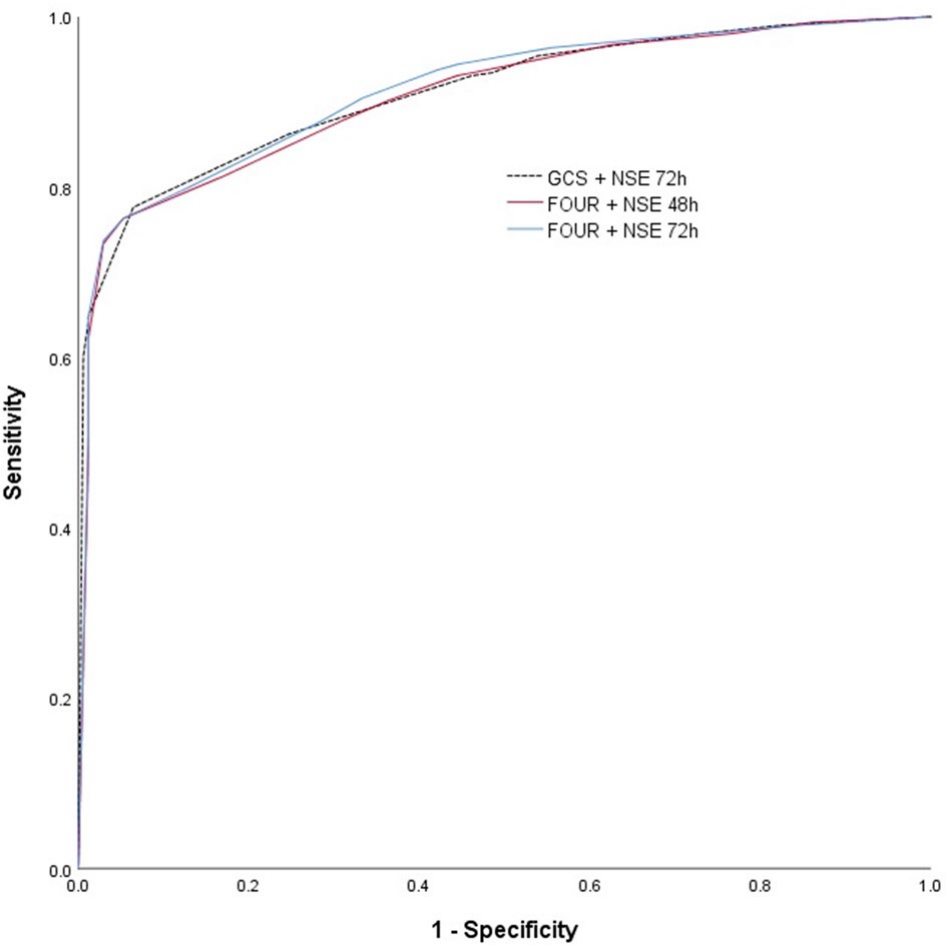
Comparison of the receiver operating characteristic curves in combinations of categorized predictors. AUC for the combination of categorized FOUR score and categorized NSE 72 h: 0.912 (criteria > 11, sensitivity = 76.3%, specificity = 94.7%); for the combination of categorized FOUR score and categorized NSE 48 h: 0.906 (criteria > 11, sensitivity = 76.3%, specificity = 94.7%); and for the combination of categorized GCS score and categorized NSE 72 h: 0.910 (criteria > 10, sensitivity = 77.6%, specificity = 93.6%). Comparison of ROC curves with Bonferroni correction: FOUR + NSE 72 h vs. FOUR + NSE 48 h, p = 0.453; FOUR + NSE 72 h vs. GCS + NSE 72 h, p = 0.770; FOUR + NSE 48 h vs. GCS + NSE 72 h, p = 0.660. AUC, area under the curve; GCS, Glasgow Coma Scale; FOUR, Full Outline of UnResponsiveness; NSE, neuron-specific enolase.

Table 3 and Fig. 2 show the performance data of each predictor and their combinations in predicting 6-month poor neurological outcomes. For each categorized predictor and their combinations, the area under the curve (AUC) of the categorized NSE level at 48 and 72 h, GCS score, and FOUR score were 0.879 (95% confidence interval [CI], 0.846–0.907), 0.889 (95% CI 0.858–0.916), 0.722 (95% CI 0.680–0.762), and 0.779 (95% CI 0.739–0.816), respectively (Table 3). The AUCs of the combinations of the categorized NSE level at 72 h with the categorized GCS score and FOUR score were 0.910 (95% CI 0.885–0.936) and 0.912 (95% CI 0.886–0.938), respectively. Each combination was significantly higher than the AUC value of the NSE level at 72 h alone (with GCS, ΔAUC = 0.021 [95% CI 0.004–0.038], p = 0.0153; with FOUR, ΔAUC = 0.023 [95% CI 0.003–0.043], p = 0.0257). The predictive performances of the combinations of the categorized NSE level at 48 h with the categorized GCS score and FOUR score were also higher than the performance of any variable alone (Table 3).

**Table 3:** Performance of neuron-specific enolase levels and neurologic scores for predicting neurological outcomes.

	Categorized variables						Original variables					
	Cutoff	AUC	Sensitivity	Specificity	PPV	NPV	Cutoff	AUC	Sensitivity	Specificity	PPV	NPV
GCS	> 4	0.722 (0.680–0.762)	85.9 (81.4–89.6)	56.1 (48.4–63.7)	77.7(74.5– 80.6)	69.1(62.2– 75.3)	≤ 3	0.726 (0.683–0.765)	85.9 (81.4–89.6)	56.1 (48.4–63.7)	77.7 (74.5–80.6)	69.1 (62.2–75.2)
FOUR score	> 4	0.779 (0.739–0.816)	86.5 (82.2–90.1)	60.8 (53.1–68.2)	79.7(76.4– 82.6)	71.7(65.0– 77.5)	≤ 2	0.783 (0.743–0.819)	79.9 (75.0–84.3)	70.8 (63.3–77.5)	82.9 (79.3–86.1)	66.5 (60.8–71.7)
NSE 48 h	> 1	0.879 (0.846–0.907)	79.6 (74.6–84.0)	87.7 (81.8–92.2)	92.0(88.5– 94.5)	70.7(65.8– 75.3)	> 41.5 ng/mL	0.894 (0.863–0.921)	79.3 (74.3–83.7)	89.5 (83.9–93.6)	93.0 (89.6–95.4)	70.8 (66.0–75.3)
NSE 72 h	> 1	0.889 (0.858–0.916)	80.6 (75.7–84.9)	89.5 (83.9–93.6)	93.2(89.8– 95.5)	72.2(67.2– 76.7)	> 49.3 ng/mL	0.895 (0.864–0.921)	77.0 (71.8–81.6)	94.2 (89.5–97.2)	95.9 (92.7– 97.7)	69.7 (65.1–73.9)
GCS + NSE 48 h	> 10	0.901 (0.874–0.928)	76.6 (71.5–81.3)	91.8 (86.6–95.5)	94.3 (90.9–96.5)	68.9 (64.2–73.1)	–	0.910 (0.886–0.935)	81.3 (76.4–85.5)	88.9 (83.2–93.2)	92.9 (89.5–95.2)	72.7 (67.7–77.2)
GCS + NSE 72 h	> 10	0.910 (0.885–0.936)	77.6 (72.5–82.2)	93.6 (88.8–96.7)	95.5 (92.4–97.4)	70.2 (65.5–74.4)	–	0.914 (0.890–0.938)	76.6 (71.5–81.3)	95.9 (91.7–98.3)	97.1 (94.1– 98.6)	69.8 (65.3–73.9)
FOUR score + NSE 48 h	> 11	0.906 (0.879–0.932)	76.3 (71.1–81.0)	94.7 (90.2–97.6)	96.3 (93.2–98.0)	69.2 (64.7–73.4)	–	0.912 (0.888–0.937)	79.3 (74.3–83.7)	91.8 (86.6–95.5)	94.5 (91.2– 96.6)	71.4 (66.6–75.7)
FOUR score + NSE 72 h	> 11	0.912 (0.886–0.938)	76.3 (71.1–81.0)	94.7 (90.2–97.6)	96.3 (93.2–98.0)	69.2 (64.7–73.4)	–	0.917 (0.893–0.940)	77.3 (72.2–81.9)	97.1 (93.3–99.0)	97.9 (95.2–99.1)	70.6 (66.1–74.8)

AUC: area under the curve; PPV: positive predictive value; NPV: negative predictive value; GCS: Glasgow Coma Scale; FOUR: Full Outline of UnResponsiveness; NSE: neuron-specific enolase.

The receiver operating characteristic (ROC) curves were also analyzed to determine the predictive performance of the original variables (continuous or nominal variables): serum NSE level, neurological examination scores, and their combinations. The results are presented in Table 3.

## Discussion

This study showed that the combination of initial neurological examination and serum NSE assay is superior to either test alone for predicting poor neurological outcomes 6 months after cardiac arrest. We also demonstrated the feasibility of NSE values using weighted categorical values when combined with another prognostication tool.

Current international guidelines do not recommend the use of NSE level as the sole predictor in the prognostic assessment of patients with cardiac arrest^5^. Efforts have been made to combine the NSE level with another prognosticator for the prediction of neurological outcomes in patients with OHCA. Lee et al. reported that a combination of NSE level and quantitative parameters in brain CT improved prognostic performance when compared with either component alone in predicting poor neurological outcomes in patients with OHCA^17^. Ryoo et al. recently combined the NSE level at 48 h and lactate level measured after ROSC in the prognostic assessment of patients^18^; the combination yielded no synergic effect. Luescher et al. observed that NSE level measured on the third day following cardiac arrest significantly improved the clinical risk scores for outcome predictions^19^. Pfeifer et al. reported that the combination of NSE level at 72 h after cardiopulmonary resuscitation (CPR) and GCS score allows for a more reliable prediction of outcomes^20^. Our sample size of 475 patients was relatively larger than those of the aforementioned studies (97–336 patients), and the cutoff NSE levels used in the aforementioned studies also varied (41.8–82.5 ng/mL). In addition, in contrast to our study, the NSE level used in the combinations was treated as a continuous variable. In our study, weighted categorical values were used in combination with other predictors instead of absolute serum NSE cutoff values. Although the areas under the ROC (AUROCs) of the original continuous NSE values at 72 h and categorized NSE values at 72 h differed slightly (0.895 versus 0.889, p = 0.254), synergism through combination was maintained. In this study, the NSE assays performed at the participating hospitals were not uniform. Given the variability of the cutoff NSE value across previous studies and differences in NSE assay methods across institutions, it might be helpful to categorize the NSE value when applying it to the prognostication of post-resuscitation patients in clinical practice. Clinicians might already be familiar with ways to select and weigh distinct variables and to convert them to scores, as this concept has been incorporated into a risk prediction model in intensive care units (e.g., the Simplified Acute Physiology Score, Acute Physiology, and Chronic Health Evaluation score). In addition, several scoring models have been suggested for predicting the neurological outcomes of patients with cardiac arrest^6,14^.

Based on previous studies, no clinical neurological signs can reliably predict poor outcomes at < 24 h after cardiac arrest^2^. International guidelines do not recommend the use of neurological examinations in the early phases following ROSC^8^. TTM is usually administered to patients who are non-responsive to verbal commands or patients with coma after ROSC. Comatose mentality is defined as the state in which an individual has a GCS score of ≤ 8. Therefore, grades in each element of the GCS or FOUR scale might vary among patients with cardiac arrest who underwent TTM. Several studies have reported that the motor grade of GCS measured early after ROSC is associated with neurological prognosis^7,21,22^. The higher the patient's motor grade after ROSC, the better the patient’s neurological prognosis. Some studies have combined the initial FOUR score with other prognostic tools^6,8,9^. Youn et al. reported that combining initial brain stem reflex FOUR score with continuous EEG patterns is superior to any individual test in predicting survival after cardiac arrest^8^. Their subsequent study also revealed improved prognostic performance when the initial FOUR score was combined with the parameters of brain CT and continuous EEG patterns ^9^. In contrast with previous studies that used scores of only one element of the GCS and FOUR scale, we used the sum of the scores in each element of the GCS and FOUR scale. The total score might better reflect neurological prognosis than the individual elements of the neurological grading scales. In our study, the discriminative power of the total score of the two neurological grading scales was higher than that of their individual elements.

The FOUR scale includes additional information that is not assessed in the GCS, such as brainstem reflexes, visual tracking, breathing patterns, and respiratory drive^6,8^. Due to these differences between the FOUR scale and GCS, the predictive power of the FOUR score for poor outcomes might be superior to that of the GCS score. However, whether the advantages of the FOUR score remain when each neurological grading scale is combined with the NSE level has not been shown. This result might be attributed to the use of a small number of categories derived for both the neurological grading scales in the combination process, the similar AUC values of the element with the highest AUC in both coma scales, as well as the moderate association between brainstem reflex and motor response in the FOUR scale (r = 0.545). Few studies have compared the use of the GCS score and FOUR score to predict the prognosis of patients with cardiac arrest. Fugate et al. found that the FOUR score is an accurate predictor of outcomes in survivors of cardiac arrest, similar to the GCS score^23^. According to Weiss et al., the FOUR score provides a more accurate prognosis of poor neurological outcomes in patients with OHCA than does the GCS score^24^. However, Topcuoglu et al. reported results that conflict with those of Weiss et al.^25^.

Our study has some limitations. First, the possibility of selection bias cannot be ruled out because approximately 30% of the participating hospitals did not measure NSE levels during neurological prognostication. This could also limit the generalizability of the research results. However, baseline characteristics and neurological outcomes were similar when comparing included and excluded patients in the final analysis (Supplementary Table S3). Second, as the NSE level measured with the Roche method is reportedly 1.3 times higher than the NSE level measured using the Diasorin method^26^, our study is also limited by differences in serum NSE testing methods. Furthermore, information on hemolysis was not included in the registry. As NSE levels are influenced by hemolysis (a potential disadvantage of NSE), the lack of these data may also have diminished the validity of our findings. Third, although we excluded patients who elected WLST from the analysis, the results of the neurological examination and NSE level might be prone to the risk of self-fulfilling prophecy because treating physicians could not be blinded. This could have influenced treatment aggressiveness in patients who did not have WLST but were determined to have a poor prognosis. However, information on treatment aggressiveness of individual patients was not included in our registry. Fourth, the use of sedative drugs and neuromuscular blocking agents could have influenced the neurological examination. However, our registry did not include information regarding the timing, dosage, and duration of such pharmacotherapies administered after arrival at the hospital. Finally, the scorecard used in this study may need to be further refined using a more robust sample size, and external validation may be required.

In conclusion, the combination of categorized serum NSE levels and initial neurological examination improved the prediction of neurological outcomes 6 months after cardiac arrest compared with either test alone. Further studies are warranted to validate these findings.

## Methods

### Data resources and study setting

This was a retrospective analysis of data collected prospectively by the KORHN-pro registry from November 2015 to December 2018. Data were collected from patients who were admitted to 22 hospitals across South Korea. This study was approved by all participating hospitals, including the Institutional Review Board of Samsung Changwon Hospital (IRB No. SCMC 2015-10-055-099) and registered at a clinical trial registry platform (ClinicalTrials.gov Identifier: NCT02827422). Informed written consent was obtained from all patients enrolled in this study. This study followed the Strengthening the Reporting of Observational Studies in Epidemiology guidelines and checklist^27^ and complied with the tenets of Declaration of Helsinki.

The Korean emergency medical service (EMS) system is operated exclusively by the National Fire Agency. EMS providers must continue resuscitation efforts until ROSC is achieved at the scene or until arrival at the hospital. The EMS level is basic-to-intermediate, and the use of sedatives by EMS providers is not permitted. All emergency departments generally provide advanced cardiac life support, acute cardiac care, and post-resuscitation care, including the administration of sedatives and neuromuscular blockers. The enrolled patients underwent TTM according to the protocol of each hospital.

The process for data management of the KORHN-pro registry has been described in several previous studies^28–30^. Prehospital, resuscitation, and outcome data were collected according to the Utstein style. The principal investigator from each participating hospital reviewed the hospital records of OHCA survivors who underwent TTM. Neurological outcomes at discharge and at 1 and 6 months after ROSC were investigated by researchers who were blinded to patient data. Neurological outcomes were evaluated using a telephone survey or face-to-face interview with the surviving discharged patients or their relatives. Three clinical research associates monitored the data and assessed their quality by sending queries to the investigators. Finally, a data manager examined the data and decided whether the records were acceptable or required revision.

### Study population

The study included all patients with OHCA aged > 18 years, who were treated with TTM. The exclusion criteria were as follows: (1) confirmation of hemorrhagic or ischemic stroke as the cause of cardiac arrest, (2) cerebral performance category (CPC) of 3 or 4 before cardiac arrest, (3) body temperature < 30 °C upon arrival, (4) non-provision of post-resuscitation care, including TTM, (5) meaningful response to verbal commands following ROSC, (6) non-measurement of serum NSE level at 48 or 72 h after ROSC, (7) non-assessment of FOUR score or GCS score after ROSC, (8) initial GCS score > 8, (9) WLST, and (10) unknown neurological outcome at 6 months.

### Data collection and endpoint

All data were extracted from the web-based registry. The variables investigated in this study were as follows: age, sex, medical history, place of cardiac arrest (public, non-public), witnessed cardiac arrest, bystander CPR, time from collapse to ROSC, initial monitored rhythm (shockable, non-shockable), prehospital defibrillation, causes of arrest (cardiac, non-cardiac), initial serum lactate level measured after ROSC, GCS score and FOUR score obtained within 1 h of ROSC, target temperature of TTM (< 35 °C, ≥ 35 °C), serum NSE levels measured at 48 and 72 h after ROSC, and CPC scores at 1 and 6 months after cardiac arrest. The total GCS score of intubated patients was calculated by assigning one point for verbal response. The neurological outcomes were dichotomized as good (CPC 1 or 2) or poor (CPC 3 through 5). The primary outcome was poor neurological outcome at 6 months after cardiac arrest.

NSE levels at 24, 48, and 72 h after ROSC were entered into the registry. Of the 22 sites, 16 sites provided data for this biomarker. Two different NSE measurement instruments were used at the 16 sites. Hemolysis index was not included separately in the registry data. The NSE levels at 24, 48, and 72 h were entered into the registry for 575 (41.9%), 620 (45.2%), and 565 patients (41.2%), respectively; NSE measurements were available at all three time points for 349 (25.4%) patients. Only patients whose NSE levels were measured at 48 and 72 h were included in the analysis because these time points are known to be associated with the highest sensitivities and specificities^10^.

### Statistical analyses

Continuous variables are expressed as means with standard deviations or as medians and IQRs. Categorical variables are expressed as numbers and percentages. Demographic and clinical characteristics were compared between groups with good and poor neurological outcomes using the Student’s t-test, Mann–Whitney U test, Chi-squared test, or Fisher’s exact test, as appropriate.

Multivariate analyses were performed to identify independent predictors of poor neurological outcomes after adjusting for potential confounders. All variables shown in Table 1 were included in the multivariate model. NSE levels at 48 and 72 h after ROSC and GCS score and FOUR score obtained within 1 h of ROSC were confirmed for independent variables in multivariate analyses. Continuous variables were then converted to categorical variables by rounding up or down, whichever was appropriate after reaching the cutoff, using the R software optimal binning method (“smbinning” package), based on the reference variable of neurological outcomes at 6 months. The odds ratio (OR) and beta-coefficient of these variables in the unadjusted analyses were used to derive the scorecard. Weighted scores were assigned an integer value based on the relative magnitude of the OR and beta-coefficient with fixed point to double odds of 1.5. Scores were then adjusted for each category of the NSE level, GCS score, and FOUR score to ensure that total scores increased correspondingly with categories of predicted probabilities. After applying a weighted score for each category of the NSE level, GCS score, and FOUR score, we determined the AUC of each categorized variable using the ROC curve analysis. The AUC of the combination of the categorized NSE level and categorized FOUR score or GCS score was then determined from ROC curves of sums of each weighted score. ROC curve analysis was also used to determine the predictive performance of the original variables (continuous variables or nominal variables): serum NSE level, neurological examination scores, and their combinations. Comparisons of the AUROC curves were performed as recommended by DeLong et al. Statistical analyses were conducted using SPSS 24.0 (SPSS Inc., Chicago, IL, USA), R software version 3.5.2, and MedCalc 15.2.2 (MedCalc Software, Mariakerke, Belgium). A two-sided p-value of < 0.05 was considered statistically significant.

## Supplementary Information

Supplementary Information.

## Data Availability

Datasets generated and analysed during the current study are available from the corresponding author on reasonable request.
