# Supplementary material for: Combination of neuron-specific enolase measurement and initial neurological examination for the prediction of neurological outcomes after cardiac arrest
Source: Sci Rep. 2021 Jul 23;11:15067. doi: 10.1038/s41598-021-94555-0 (PMC8302684; doi:10.1038/s41598-021-94555-0)
Supplement: Supplementary file 1 — Supplementary Information. [file 41598_2021_94555_MOESM1_ESM.pdf]

Combination of Neuron-Specific Enolase Measurement and Initial Neurological Examination for the Prediction of Neurological Outcomes after Cardiac Arrest

Jae Hoon Lee [1], Yong Hwan Kim [2],* Jun Ho Lee [2], Dong Woo Lee [2], Seong Youn Hwang [2], Chun Song Youn [3], Ji-Hoon Kim [4], Min Seob Sim [5], Kyung Woon Jeung [6]

[1]Department of Emergency Medicine, Dong-A University College of Medicine, Busan, South Korea; leetoloc@dau.ac.kr

[2]Department of Emergency Medicine, Samsung Changwon Hospital, Sungkyunkwan University School of Medicine, Changwon, South Korea; galjoun@hanmail.net (J.H.L.), calmriver@hanmail.net (D.W.L.), 3syellow@naver.com (S.Y.H.)

[3]Department of Emergency Medicine, Seoul St. Mary's Hospital, College of Medicine, The Catholic University of Korea, Seoul, South Korea; ycs1005@catholic.ac.kr

[4]Department of Emergency Medicine, Bucheon St. Mary's Hospital, College of Medicine, The Catholic University of Korea, Seoul, South Korea; intimator@catholic.ac.kr

[5]Department of Emergency Medicine, Samsung Medical Center, Sungkyunkwan University School of Medicine, Seoul, South Korea; coldco2@naver.com

[6]Department of Emergency Medicine, Chonnam National University, Chonnam National University Hospital, Gwangju, South Korea; neoneti@hanmail.net

*Address for Correspondence:

Yong Hwan Kim

Department of Emergency Medicine, Samsung Changwon Hospital, Sungkyunkwan University School of Medicine, 158, Paryong-ro, Masanhoewon-gu, Changwon-si, Gyeongsangnam-do 630-723, South Korea, E-mail address: suka1212@hanmail.net

Supplementary Table S1. Association between strata of the neuron-specific enolase level and initial neurological examination data and 6-month neurological outcomes

**Variable**	**Good outcome (n = 171)**	**Poor outcome (n = 304)**	**p-value**
**GCS score**			< 0.001
**3**	75 (22.3)	261 (77.7)	
**4 and 5**	46 (57.5)	34 (42.5)	
**6–8**	50 (84.7)	9 (15.3)	
**FOUR score**			< 0.001
**0 and 1**	40 (16.1)	208 (83.9)	
**2 and 3**	27 (32.9)	55 (67.1)	
**4–6**	61 (64.2)	34 (35.8)	
**≥ 7**	43 (86.0)	7 (14.0)	
**NSE level at 48 h, ng/mL**			< 0.001
**NSE < 21**	77 (74.8)	26 (25.2)	
**21 ≤ NSE < 40**	73 (67.0)	36 (33.0)	
**41 ≤ NSE < 80**	19 (26.4)	53 (73.6)	
**81 ≤ NSE**	2 (1.0)	189 (99.0)	

**NSE level at 72 h, ng/mL**			< 0.001
**NSE < 21**	100 (75.8)	32 (24.2)	
**21 ≤ NSE < 40**	53 (66.2)	27 (33.7)	
**41 ≤ NSE < 80**	16 (25.0)	48 (75.0)	
**81 ≤ NSE value**	2 (1.0)	197 (99.0)	

GCS, Glasgow Coma Scale; FOUR, Full Outline of UnResponsiveness; NSE, neuron-specific enolase

Supplementary Table S2. The scorecard for neurological examinations and neuron-specific enolase levels

**Characteristics**	**Coefficient**	**Odds ratio**	**95% confidence interval**		**Weighted score**
			Low	Upper	
**GCS score**					
**6–8**	Reference				0
**4 and 5**	1.413	4.106	1.778	9.481	4
**3**	2.962	19.333	9.089	41.122	9
**FOUR score**					
**≥ 7**	Reference				0
**4–6**	1.231	3.424	1.389	8.440	4
**2 and 3**	2.527	12.513	4.976	31.465	7
**0 and 1**	3.464	31.943	13.416	76.057	10
**NSE 48 h, ng/mL**					
**NSE < 21**	Reference				0
**21 ≤ NSE < 41**	0.379	1.460	.804	2.654	1
**41 ≤ NSE < 81**	2.112	8.261	4.155	16.425	6
**81 ≤ NSE**	5.634	279.865	64.837	1208.017	16
**NSE 72 h, ng/mL**					
**NSE < 21**	Reference				0
**21 ≤ NSE < 41**	0.465	1.592	0.864	2.933	1
**41 ≤ NSE < 81**	2.238	9.375	4.694	18.725	6
**81 ≤ NSE**	5.729	307.812	72.299	1310.516	17

GCS, Glasgow Coma Scale; FOUR, Full Outline of UnResponsiveness; NSE, neuron-specific enolase

Supplementary Table S3. Comparison between enrolled and unenrolled patients

Variables	Enrolled patients N = 475	Unenrolled patients* N = 589	p-value
**Age (years)**	59 (48–69)	58 (46–69)	0.090
**Sex, male**	346 (72.8)	405 (68.8)	0.146
**Initial shockable rhythm**	164 (34.5)	229 (38.9)	0.144
**Prehospital defibrillation**	183 (38.5)	243 (41.3)	0.366
**Witnessed arrest**	332 (69.9)	424 (72.0)	0.455
**Bystander CPR, yes**	291 (61.3)	383 (65.0)	0.205
**Cardiac etiology**	298 (62.7)	368 (62.5)	0.931
**Cardiac arrest at public place**	232 (48.8)	272 (46.2)	0.387
**Time from collapse to ROSC, minutes**	27.9 (15.9–41.0)	26.0 (16.0–39.0)	0.163
**Target temperature, < 35°C**	362 (76.2)	485 (82.3)	0.014
**Initial lactate level, mmol/L**	9.2 (5.7–12.5)	9.4 (5.6–12.4)	0.590
**CPC at 6 months, 1 or 2**	171 (36.0)	225 (38.2)	0.460

Data are reported as n (%) or medians (interquartile ranges).

*For the 898 patients not enrolled in the present study, exclusion criteria were as follows: unknown neurological outcome at 6 months (n = 32), initial Glasgow Coma Scale score > 8 (n = 14), withdrawal of life-sustaining therapy decision (n = 36), and death within 72 h after ROSC (n = 227).

CPR, cardiopulmonary resuscitation; ROSC, return of spontaneous circulation; CPC, cerebral performance category
